# Preoperative middle meningeal artery embolization is associated with reduced reoperation rates in chronic subdural hematoma

**DOI:** 10.1177/15910199251372487

**Published:** 2025-09-02

**Authors:** Hamza Adel Salim, Waseem Shehadeh, Orabi Hajjeh, Adam A. Dmytriw, Huanwen Chen, Muhammed Amir Essibayi, Nimer Adeeb, Ahmed Msherghi, Marco Colasurdo, Ajay Malhotra, Vivek S Yedavalli, Dheeraj Gandhi, Max Wintermark, Dhairya A Lakhani

**Affiliations:** 1 Department of Neuroradiology, 4002 MD Anderson Medical Center, Houston, TX, USA; 2 Department of Neuroradiology, Rockefeller Neuroscience Institute, West Virginia University, Morgantown, WV, USA; 3 Neuroendovascular Program, Massachusetts General Hospital, 1813 Harvard University, Boston, MA, USA; 4 Neurovascular Centre, Departments of Medical Imaging and Neurosurgery, St Michael's Hospital, Toronto, ON, Canada; 5 Division of Neurointerventional Surgery, Department of Neurosurgery, 21668 University of Maryland Medical Center, Baltimore, MD, USA; 6 Department of Neurological Surgery and Montefiore-Einstein Cerebrovascular Research Lab, Montefiore Medical Center, 2013 Albert Einstein College of Medicine, Bronx, NY, USA; 7 Department of Neurosurgery and Interventional Neuroradiology, 5779 Louisiana State University, Shreveport, LA, USA; 8 Department of Interventional Radiology, 6684 Oregon Health and Science University, Portland, OR, USA; 9 Department of Radiology, 25047 Yale New Haven Hospital, New Haven, CT, USA; 10 Department of Radiology, Division of Neuroradiology, Johns Hopkins Medical Center, Baltimore, MD, USA

**Keywords:** Middle meningeal artery embolization, chronic subdural hematoma, preoperative embolization, hematoma recurrence, endovascular neurosurgery

## Abstract

**Background:**

Middle meningeal artery embolization (MMAE) has recently emerged as a promising adjunctive therapy to surgical evacuation for patients with chronic subdural hematoma (cSDH). However, the optimal timing of MMAE relative to surgery remains poorly defined. Therefore, this large retrospective cohort study aimed to assess the impact of MMAE timing (preoperative vs. postoperative) on 6-month outcomes in patients with cSDH, focusing on rates of repeat surgery and mortality. We hypothesized that preoperative MMAE would be associated with lower rates of reoperation compared to postoperative MMAE.

**Methods:**

Adult patients with nontraumatic cSDH who underwent surgery with adjunctive MMAE were identified using ICD-10 codes from the TriNetX database. A 1:1 propensity score matching approach was used to balance baseline characteristics between groups. The primary outcomes were repeat surgery and all-cause mortality within 6 months.

**Results:**

A total of 338 matched patients (*n* = 338; 169 in each group) were included in the final analysis. Preoperative MMAE was associated with significantly lower odds of repeat surgery compared to postoperative MMAE (7.1% vs. 17.8%; OR 0.35, *p* = 0.003). No significant difference was observed in 6-month all-cause mortality between the groups.

**Conclusion:**

Preoperative MMAE is associated with reduced odds of repeat surgery compared to postoperative MMAE at 6 months. These findings support consideration of MMAE timing in surgical planning. Further prospective studies are warranted to validate these results.

## Introduction

The incidence of chronic subdural hematoma (cSDH) is rising, particularly among the elderly, and is associated with significant morbidity and mortality.^1–4^ Conventionally, the primary treatment option for cSDH is surgical drainage for patients without major comorbidities or antithrombotic use. However, recurrence is common, with many patients requiring repeat surgery.^5–7^

Emerging evidence suggests that the formation of fragile neovessels within the membranes of cSDH is a key driver of hematoma recurrence and expansion, due to their tendency to rupture. This has led to growing interest in middle meningeal artery embolization (MMAE), which devascularizes these neovessels and reducing the risk for repeat hemorrhage.^4,5,8–11^ Several real-world studies and clinical trials have shown that combining surgery with adjunctive MMAE leads to better outcomes than surgery alone, including reduced mortality and lower reoperation rates. However, there is no clear consensus on when MMAE should be performed in relation to the surgery.^8,9,12,13^ It is believed that many serious surgical complications, such as MMA injury, pseudoaneurysm formation, and iatrogenic MMA-to-middle meningeal vein fistulae, are linked to manipulation of MMA. Therefore, it can be speculated that performing MMAE before surgery may reduce these serious risks by addressing the vascular pathology in advance, potentially improving outcomes compared to postoperative MMAE. However, the optimal timing of MMAE relative to surgery remains poorly defined.^7–9,12,14^ Clinical trials have been inconsistent: STEM and MAGIC-MT used preoperative MMAE, EMPROTECT used it postoperatively, while EMBOLISE and MEMBRANE included either preoperative or postoperative MMAE approaches. As such, there is a lack of robust evidence directly comparing MMAE timing strategies.^14^

Therefore, this large retrospective cohort study aimed to assess the impact of MMAE timing (preoperative vs. postoperative) on 6-month outcomes in patients with cSDH, specifically focusing on rates of repeat surgery and mortality. We hypothesize that preoperative MMAE is associated with lower rates of reoperation compared to postoperative MMAE.

## Methods

### Study design and data source

This was a retrospective cohort study utilizing de-identified, real-world patient data obtained from the TriNetX (https://trinetx.com/), a federated network of electronic health records. Adult patients diagnosed with nontraumatic cSDH who underwent surgery with adjunctive MMAE were included. Diagnoses and procedures were identified using ICD-10-CM and ICD-10-PCS codes as shown in Supplemental Table 1.

### Exposure and grouping

The primary exposure was the timing of MMAE in relation to the surgical evacuation of cSDH. Patients were divided into two groups: those who underwent MMAE before surgery (preoperative MMAE) and those who received MMAE after surgery (postoperative MMAE). The timing was defined based on procedural date relationships within each patient's health record. The index event for each patient was the first qualifying surgery, with the MMAE procedure occurring either within 1 month before or within 1 month after surgery.

### Outcomes

The outcomes included the rate of repeat surgical evacuation, and all-cause mortality within 6 months. The procedural codes used to define outcome measures are described in Supplemental Table 1.

### Statistical analysis

Categorical variables were compared using chi-square or Fisher's exact tests, and continuous variables using independent *t*-tests. To reduce confounding, 1:1 nearest-neighbor propensity score matching without replacement was performed. Matching variables included age, sex, race/ethnicity, hypertension, diabetes mellitus, ischemic heart disease, cerebral infarction, obesity, and use of anticoagulants or antiplatelet agents. The balance between groups was evaluated using absolute standardized differences (ASDs), with values <0.1 considered acceptable. Results were reported as odds ratios (OR) with corresponding 95% confidence intervals (CIs). A two-sided *p*-value <0.05 was considered statistically significant. The missing data were excluded for variables included in the propensity score matching and for outcome analyses.

## Results

### Baseline characteristics

A total of 790 patients with cSDH who underwent surgery with adjunctive MMAE were identified. Among them, 170 patients received preoperative MMAE, while 620 underwent postoperative MMAE. After propensity score matching, 338 patients were included in the final analysis, with 169 patients in each group (preoperative and postoperative MMAE). Baseline demographics and clinical comorbidities were well balanced between the two groups, with all ASDs below 0.1, indicating appropriate covariate balance (Table 1). Baseline characteristics prior to matching are presented in Supplemental Table 2.

**Table 1.** Baseline characteristics after propensity score matching (*N* = 169 per group).

Characteristic	MMAE before surgery (*n* = 169)	MMAE after surgery (*n* = 169)	*p*-Value	ASD
Age (mean ± SD)	72.7 ± 12.1	73.3 ± 11.7	0.629	0.053
Gender				
Female	40 (23.7%)	43 (25.4%)	0.705	0.041
Male	123 (72.8%)	123 (72.8%)	1.000	<0.001
Race				
White	121 (71.6%)	118 (69.8%)	0.720	0.039
Black or African American	18 (10.7%)	18 (10.7%)	1.000	<0.001
Asian	14 (8.3%)	17 (10.1%)	0.572	0.062
Hispanic or Latino	13 (7.7%)	14 (8.3%)	0.841	0.022
Not Hispanic or Latino	139 (82.2%)	140 (82.8%)	0.886	0.016
Comorbidities				
Hypertension	141 (83.4%)	142 (84.0%)	0.883	0.016
Diabetes mellitus	60 (35.5%)	50 (29.6%)	0.246	0.127
Stroke	26 (15.4%)	26 (15.4%)	1.000	<0.001
Ischemic heart disease	63 (37.3%)	58 (34.3%)	0.571	0.062
Overweight and obesity	42 (24.9%)	46 (27.2%)	0.620	0.054
Medications (prior use)				
Anticoagulant use	152 (89.9%)	152 (89.9%)	1.000	<0.001
Antiplatelet use	75 (44.4%)	80 (47.3%)	0.585	0.059

MMAE: middle meningeal artery embolization; ASD: absolute standardized difference.

### MMAE timing and outcomes

Following propensity score matching, preoperative MMAE was associated with significantly lower odds of requiring repeat surgical evacuation compared to postoperative MMAE (7.1% vs. 17.8%; OR 0.35, 95% CI 0.18–0.72, *p* = 0.003) (Table 2). There was no significant difference in all-cause mortality between the two groups (10.1% vs. 10.1%; OR 1.00, 95% CI 0.49–2.03, *p* = 1.000) (Table 2).

**Table 2.** Clinical outcomes after propensity score matching (*N* = 169 per group).

Outcome	MMAE before surgery (*n* = 169)	MMAE after surgery (*n* = 169)	OR (95% CI)	*p*-Value
Repeat surgery	12 (7.1%)	30 (17.8%)	0.35 (0.18–0.72)	0.003
Mortality	17 (10.1%)	17 (10.1%)	1.00 (0.49–2.03)	1.000

MMAE: middle meningeal artery embolization; OR: odds ratio; CI: confidence interval.

## Discussion

In this large retrospective cohort study using real-world, propensity score-matched data, we investigated the clinical impact of the timing of MMAE relative to surgical evacuation in patients with cSDH. Our findings indicate that preoperative MMAE is associated with significantly lower odds of requiring repeat surgery compared to postoperative MMAE (7.1% vs. 17.8%; OR 0.35, 95% CI 0.18–0.72, *p* = 0.003). This substantial reduction in repeat surgical interventions, with comparable all-cause mortality between groups, suggests that performing adjunctive MMAE prior to surgery may be more beneficial than after surgery, potentially by reducing intraoperative complications related to MMA injury. Some institutions have adopted a pilot strategy of performing MMAE prior to surgical drainage. This approach is based on the understanding that many complications are often driven by MMA pathology. Conversely, postoperative embolization may occur in certain cases due to logistical delays, evolving clinical indications, or institutional variability in workflow. Unfortunately, procedural timing decisions are not captured in the database, but future studies should explore this further.

Most of the perioperative complications and serious rebleeds result from MMA pseudoaneurysm, MMA-to-middle meningeal vein fistulae, or direct injury to fragile neovasculature during surgical manipulation.^15–17^ Damage to the fragile neovessels adds to a vicious cycle of rebleeding and hematoma expansion.^3,9,18–20^ Hence, performing MMAE before surgery may be reasonable, as it may reduce vascular supply to these vulnerable vessels, potentially preventing rebleeding and improving surgical outcomes. Additionally, it may leads to membrane devascularization, which could lower the rate of its injury during surgical manipulation.^21^ Hence, in our study we observed lower rates of needing repeat surgery in patients who had preoperative MMAE compared to postoperative MMAE. However, our findings contrast with a recent study that found no significant difference in reoperation rates, regardless of whether MMAE was done before or after surgery MMAE (8% (4 of 48) vs. 4% (4 of 93)).^14^ However, their sample was smaller, potentially limiting statistical power. Meanwhile, no other previous studies have specifically addressed this association.

Our study has several strengths, including the large sample size and real-world data. To our knowledge, this is the first real-world propensity score-matched analysis comparing outcomes of preoperative and postoperative MMAE. However, we acknowledge several limitations. First, as a retrospective analysis based on electronic health records, the study is subject to residual confounding despite the use of propensity score matching. Second, the timing of embolization (before vs. after surgery) may still reflect clinical judgment influenced by patient condition or logistical factors that were not fully captured in the dataset. It is also possible that the preoperative MMAE group inadvertently included patients who were initially intended to be managed with MMAE alone but later required rescue surgery. As a result, they might have had smaller hematomas compared to those in the postoperative MMAE group. Third, imaging data were not available; therefore, we could not match cohorts based on the presence of concurrent bilateral SDHs, hematoma thickness or volume, or the presence of midline shift. Finally, we were unable to assess functional status as an outcome measure. Additionally, details regarding the specific embolic materials used during MMAE were not captured in the database, which limits procedural reproducibility and interpretation of technical factors. There is a need for prospective studies assessing impact of pre- and postoperative MMAE on outcomes.

## Conclusion

Preoperative MMAE is associated with lower odds of repeat surgery rates at 6 months compared to postoperative MMAE. These findings provide important evidence supporting the consideration of MMAE timing in surgical planning for cSDH. Further prospective studies are needed to validate these results and guide clinical recommendations on the optimal timing of MMAE in the management of cSDH.

## Supplemental Material

sj-docx-1-ine-10.1177_15910199251372487 - Supplemental material for Preoperative middle meningeal artery embolization is associated with reduced reoperation rates in chronic subdural hematoma Supplemental material, sj-docx-1-ine-10.1177_15910199251372487 for Preoperative middle meningeal artery embolization is associated with reduced reoperation rates in chronic subdural hematoma by Hamza Adel Salim, Waseem Shehadeh, Orabi Hajjeh, Adam A. Dmytriw, Huanwen Chen, Muhammed Amir Essibayi, Nimer Adeeb, Ahmed Msherghi, Marco Colasurdo, Ajay Malhotra, Vivek S Yedavalli, Dheeraj Gandhi, Max Wintermark and Dhairya A Lakhani in Interventional Neuroradiology
