# Supplementary material for: Preoperative middle meningeal artery embolization is associated with reduced reoperation rates in chronic subdural hematoma
Source: Interv Neuroradiol. 2025 Sep 2:15910199251372487. Online ahead of print. doi: 10.1177/15910199251372487 (PMC12405205; doi:10.1177/15910199251372487)
Supplement: sj-docx-1-ine-10.1177_15910199251372487 - Supplemental material for Preoperative middle meningeal artery embolization is associated with reduced reoperation rates in chronic subdural hematoma [file sj-docx-1-ine-10.1177_15910199251372487.docx]

**Supplementary Table 1: ICD and ICD-10-PCS Codes Used in This Study**

| Group | Code | Definition |
| --- | --- | --- |
| Study Population Definition | ICD-10-CM: I62.03 | Nontraumatic chronic subdural hemorrhage |
| Procedure Definition: MMAE | ICD-10-PCS: 03LG3DZ | Occlusion of intracranial artery with intraluminal device, percutaneous |
| Procedure Definition: Surgical Evacuation | ICD-10-PCS: 00C43ZZ | Extirpation of matter from intracranial subdural space, percutaneous approach |
|  | ICD-10-PCS: 00C44ZZ | Extirpation of matter from intracranial subdural space, endoscopic approach |
|  | ICD-10-PCS: 00C40ZZ | Extirpation of matter from intracranial subdural space, open approach |
|  | ICD-10-PCS: 00943ZZ | Drainage of subdural space, percutaneous approach |
|  | ICD-10-PCS: 00944ZZ | Drainage of subdural space, endoscopic approach |
|  | ICD-10-PCS: 009430Z | Drainage with device, subdural space, percutaneous |
|  | ICD-10-PCS: 009440Z | Drainage with device, subdural space, endoscopic |
|  | ICD-10-PCS: 00940ZZ | Drainage of subdural space, open approach |
|  | ICD-10-PCS: 009400Z | Drainage with device, subdural space, open approach |
| Outcome Definition: Mortality | EHR Event | Death as recorded in electronic health record |
| Outcome Definition:  Repeat Surgery | Same PCS codes | Reappearance of surgical procedure code post-index surgery |

Supplementary Table 2: Baseline Characteristics Before Propensity Score Matching (PSM)

| Characteristic | MMAE Before Surgery (n=170) | MMAE After Surgery (n=620) | p-value | ASD |
| --- | --- | --- | --- | --- |
| Age (mean ± SD) | 72.8 ± 12.2 | 71.9 ± 12.3 | 0.425 | 0.069 |
| Gender | | | | |
| Female | 40 (23.5%) | 144 (23.2%) | 0.934 | 0.007 |
| Male | 123 (72.4%) | 467 (75.3%) | 0.430 | 0.068 |
| Race | | | | |
| White | 121 (71.2%) | 420 (67.7%) | 0.393 | 0.075 |
| Black or African American | 18 (10.6%) | 68 (11.0%) | 0.888 | 0.012 |
| Asian | 14 (8.2%) | 60 (9.7%) | 0.568 | 0.051 |
| Hispanic or Latino | 13 (7.6%) | 40 (6.5%) | 0.581 | 0.047 |
| Not Hispanic or Latino | 139 (81.8%) | 495 (79.8%) | 0.576 | 0.049 |
| Comorbidities | | | | |
| Hypertension | 142 (83.5%) | 478 (77.1%) | 0.071 | 0.162 |
| Stroke | 27 (15.9%) | 91 (14.7%) | 0.696 | 0.033 |
| Ischemic heart diseases | 63 (37.1%) | 218 (35.2%) | 0.647 | 0.040 |
| Diabetes mellitus | 60 (35.3%) | 211 (34.0%) | 0.759 | 0.027 |
| Overweight and Obesity | 42 (24.7%) | 142 (22.9%) | 0.622 | 0.042 |
| Medications (prior use) | | | | |
| Anticoagulant Use | 153 (90.0%) | 370 (59.7%) | <0.001 | 0.746 |
| Antiplatelet Use | 75 (44.1%) | 208 (33.5%) | 0.011 | 0.218 |
